# Empowering future doctors: smart inhaler training for MBBS students in polluted cities

**DOI:** 10.1097/MS9.0000000000003611

**Published:** 2025-07-18

**Authors:** Muhammad Talha, Maliha Khalid, Aminath Waafira

**Affiliations:** a King Edward Medical University, Lahore, Pakistan; b Jinnah Sindh Medical University, Karachi, Pakistan; c The Maldives National University, Malé, Maldives

*To the Editor,*

Elevated urban air pollution is the basis for growing occurrences of smog asthma, which is now considerably injuring public health and urgently necessitating countermeasures. With cities facing deteriorating air quality in the year 2025, keeping asthma under control will pose even more challenges. Developing clinical MBBS training using IoT-enabled smart inhalers may thus become a game changer, with future physicians being equipped with skills to stem the tide of this rising catastrophe^[1]^. The letter emphasizes the potential of smart inhalers to combat smog asthma and proposes their integration into the medical school curricula to prepare clinicians for challenges posed by urban respiratory health. This is in line with the TITAN Guidelines on the need for transparency in AI use in healthcare^[2]^.

Asthma is a chronic disease affecting people all around the world. Many of these people suffer from air pollution-related symptoms, especially those who live in crowded urban areas. Studies indicate that 70-90% of inhalers are either misused or inconsistently used in patients with asthma and COPD, thereby thwarting therapeutic outcomes and leading to unnecessary hospitalization^[3]^. Smart inhalers, inhalers equipped with sensors and connectivity, are set to open fresh channels of asthma management. Such devices can provide objective feedback in terms of medication use, inhaler technique, and environmental trigger monitoring in real time for both patients and their clinicians. With smart inhalers, a minimum of 15% increase in adherence with medications was evidenced and a 35% reduction in COPD-related hospitalizations. Smart inhalers, equipped with sensors and connectivity, address these issues by offering objective feedback on medication adherence, inhaler technique, and environmental exposures. This engenders symptom control, fewer exacerbations, and a higher quality of life for asthma patients. Real-time feedback and reminders from smart inhalers will help bridge the care gaps that exist within an asthma system, especially in polluted urban environments^[4]^.

Smart inhalers are becoming increasingly useful in an urban setting, where the very conditions under which a smart inhaler is employed become important. Smart inhalers can now monitor when a patient uses inhalers and how often inhalers are used while also keeping track of environmental conditions during that time, for instance, air quality, pollen levels, temperature, and humidity. The data thus collected can be used to tailor treatment plans and highlight certain behavioral changes that could alleviate the risk of an attack in themselves. Recent studies report that smart inhalers enable patients to be more active in managing their symptoms and aid their interaction with healthcare providers, and this in turn has led to better bronchospasm control and adherence to therapy^[5]^. Involving the medical students in the courses about such electronic gadgets as smart inhalers will prepare them to respond appropriately to the future changes in respiratory health. The devices provide real-time feedback and reminders, which is especially advantageous in resource-poor settings where follow-up with the patients is a challenge.

There are, nevertheless, drawbacks associated with the universalization of this technology: lack of access to technology, poor health literacy, and financial constraints can equate to equity in the usage of technology. However, integrating smart inhaler technology into medical education can bridge the gap created by these very problems. Training MBBS students on the use and interpretation of data collected by IoT-enabled inhalers ensures that, when they become practicing physicians, they will be in a position to champion and implement evidence-informed asthma care. This especially resonates in urbanized settings, where pollution venturiates as the number one aggravating element for asthma. Barriers to adoption such as access and literacy should be addressed so that the benefits become equitable^[3]^.

Due to air pollution and aggravation of asthma cases, it is imperative for the medical students to learn advanced technologies like smart inhalers. In fact, through the knowledge of the apps, they can target individual data better. This digital health tool must be considered in the revision of the curriculum so that young doctors in training will be well equipped to deal with the air pollution crisis. Therefore, there is a need for policymakers and medical education in general to integrate smart inhaler training into undergraduate medical education to foster a new generation of clinicians who can fight the changing landscape of urban respiratory health.

## Data Availability

No data were generated for this manuscript.
